# Injury Patterns and Hospital Admission After Trauma Among People Experiencing Homelessness

**DOI:** 10.1001/jamanetworkopen.2023.20862

**Published:** 2023-06-29

**Authors:** Casey M. Silver, Arielle C. Thomas, Susheel Reddy, Gwyneth A. Sullivan, Rebecca E. Plevin, Hemal K. Kanzaria, Anne M. Stey

**Affiliations:** 1 Department of Surgery, Northwestern University Feinberg School of Medicine, Chicago, Illinois; 2 American College of Surgeons, Chicago, Illinois; 3 Department of Surgery, Medical College of Wisconsin, Milwaukee; 4 Department of Surgery, Rush University, Chicago, Illinois; 5 Department of Surgery, University of California, San Francisco; 6 Department of Emergency Medicine, University of California, San Francisco

## Abstract

**Importance:**

Traumatic injury is a major cause of morbidity for people experiencing homelessness (PEH). However, injury patterns and subsequent hospitalization among PEH have not been studied on a national scale.

**Objective:**

To evaluate whether differences in mechanisms of injury exist between PEH and housed trauma patients in North America and whether the lack of housing is associated with increased adjusted odds of hospital admission.

**Design, Setting, and Participants:**

This was a retrospective observational cohort study of participants in the 2017 to 2018 American College of Surgeons’ Trauma Quality Improvement Program. Hospitals across the US and Canada were queried. Participants were patients aged 18 years or older presenting to an emergency department after injury. Data were analyzed from December 2021 to November 2022.

**Exposures:**

PEH were identified using the Trauma Quality Improvement Program’s alternate home residence variable.

**Main Outcomes and Measures:**

The primary outcome was hospital admission. Subgroup analysis was used to compared PEH with low-income housed patients (defined by Medicaid enrollment).

**Results:**

A total of 1 738 992 patients (mean [SD] age, 53.6 [21.2] years; 712 120 [41.0%] female; 97 910 [5.9%] Hispanic, 227 638 [13.7%] non-Hispanic Black, and 1 157 950 [69.6%] non-Hispanic White) presented to 790 hospitals with trauma, including 12 266 PEH (0.7%) and 1 726 726 housed patients (99.3%). Compared with housed patients, PEH were younger (mean [SD] age, 45.2 [13.6] years vs 53.7 [21.3] years), more often male (10 343 patients [84.3%] vs 1 016 310 patients [58.9%]), and had higher rates of behavioral comorbidity (2884 patients [23.5%] vs 191 425 patients [11.1%]). PEH sustained different injury patterns, including higher proportions of injuries due to assault (4417 patients [36.0%] vs 165 666 patients [9.6%]), pedestrian-strike (1891 patients [15.4%] vs 55 533 patients [3.2%]), and head injury (8041 patients [65.6%] vs 851 823 patients [49.3%]), compared with housed patients. On multivariable analysis, PEH experienced increased adjusted odds of hospitalization (adjusted odds ratio [aOR], 1.33; 95% CI, 1.24-1.43) compared with housed patients. The association of lacking housing with hospital admission persisted on subgroup comparison of PEH with low-income housed patients (aOR, 1.10; 95% CI, 1.03-1.19).

**Conclusions and Relevance:**

Injured PEH had significantly greater adjusted odds of hospital admission. These findings suggest that tailored programs for PEH are needed to prevent their injury patterns and facilitate safe discharge after injury.

## Key Points

**Question:**

Is homelessness associated with hospital admission following injury?

**Findings:**

In this national cohort study of 12 266 people experiencing homelessness (PEH) from the American College of Surgeons Trauma Quality Improvement Program, PEH demonstrated significantly increased adjusted odds of hospital admission after injury compared with housed patients.

**Meaning:**

These findings suggest that potential challenges in facilitating safe discharge from the emergency department may lead to increased hospital admission after injury for PEH.

## Teaser

This cohort study evaluates whether differences in mechanisms of injury exist between people experiencing homelessness and housed trauma patients in North America and whether lack of housing is associated with increased adjusted odds of hospital admission.

## Introduction

An estimated 580 000 people experienced homelessness in the US on any given night in 2020, with increasing volume during the COVID-19 pandemic.^1^ Lack of stable housing is an important health-related social risk factor.^2,3^ People experiencing homelessness (PEH) face substantial barriers to primary care, resulting in higher rates of emergency department (ED) utilization.^4,5,6,7,8^ PEH also have increased mortality rates compared with the general population.^9,10,11^

Traumatic injury accounts for up to 28% of mortality among PEH.^12,13^ Despite the high incidence, to our knowledge, no national study of the epidemiology and management of traumatic injury among PEH has been conducted. Single-center studies have suggested increased rates of falls, burns, and assaults among PEH.^14,15,16^ However, injury mechanism can frequently vary between trauma centers and geographic regions. Further exploration of common injury mechanisms could inform injury prevention efforts for PEH. Additionally, although PEH presenting to the ED with physical and behavioral health symptoms have higher rates of hospital admission compared with housed patients, what happens to traumatically injured PEH after ED presentation has not been studied at the national level.^4,17^ ED disposition after physical trauma is particularly relevant among PEH because the lack of housing contributes to a continued risk of additional injury.

In this nationwide cohort study, we aimed to define the epidemiology of traumatic injury and subsequent hospital use among PEH compared with housed patients. Specifically, we sought to (1) characterize injury patterns among PEH sustaining traumatic injury, (2) evaluate the associations of housing status with hospital admission, and (3) conduct an a priori subanalysis of PEH compared with low-income housed patients who may experience similar inequities. We hypothesized that limited options for safe ED discharge would lead to increased hospital admission among PEH compared with both all housed and low-income housed patients.

## Methods

### Data Source

This was a retrospective observational cohort study of patients in the American College of Surgeons (ACS) Trauma Quality Improvement Project (TQIP). TQIP is a nationwide traumatic injury registry containing more than 7.5 million incident-based encounters of trauma activations at participating hospitals. Encounters were submitted voluntarily by over 750 facilities across the US and Canada. Most participating facilities were ACS-verified level I or II trauma centers, although nontrauma centers were also included. Although TQIP aims to include patients with at least 1 severe injury (an Abbreviated Injury Scale score of ≥3 in at least 1 body region), patients with less severe injuries are also included. Patient, injury, and hospital data were recorded by trained dedicated abstractors.^18^ Northwestern University’s institutional review board approved the project. Informed consent was not needed because the data were anonymous, in accordance with 45 CFR §46. This study adhered to the Strengthening the Reporting of Observational Studies in Epidemiology (STROBE) reporting guidelines.

### Study Population

Adult patients aged 18 years or older who presented following injury to participating TQIP hospitals from January 1, 2017, to December 31, 2018, were identified. We excluded 14 606 encounters with no signs of life upon presentation. Observations with missing ED discharge disposition data were excluded (50 531 observations), because this field determined the primary outcome. Encounters that left against medical advice (5328 observations) were also excluded, as their disposition was not determined by clinician decision (eFigure in Supplement 1).

Given the marked systematic differences in demographic, clinical, and injury characteristics by housing status, we defined 2 further subcohorts to address potential confounding on discharge disposition. The first was a subcohort of PEH propensity score–matched to all housed patients. The second was a subcohort of PEH compared with low-income housed patients. TQIP does not contain income data, and we used Medicaid insurance as a proxy for low income, because Medicaid eligibility is determined largely by income at or below the federal poverty limit.^19,20^

### Exposure of Interest

The exposure of interest was documented housing status. PEH were identified using TQIP’s alternate home residence variable, which is completed for individuals who do not have a temporary or permanent residence ZIP code listed on identification documents. Clinical abstractors are trained to record these patients’ ZIP code as not applicable and complete the alternate home residence variable. When patients did have a ZIP code listed, this variable was not completed and we classified them as housed. Encounters for which the alternate home residence variable was recorded as undocumented citizen or migrant worker rather than homeless were also considered to be housed.

### Outcomes

The primary outcome was hospital admission created as a binary variable (admitted vs not admitted). Hospital admission was derived from TQIP’s ED discharge disposition variable; categories included observational unit, inpatient unit, transfer, home, other facility, and deceased. Patients discharged home from the ED account for approximately 9% of records in TQIP. Patients were considered admitted if they were admitted to an observational or inpatient unit or if they were transferred to another hospital. We considered patients not admitted if their disposition was home or other facility. TQIP’s other facility category includes jail, institutional care, and mental health facilities. Although it may have been appropriate to consider individuals admitted for psychiatric care to have been admitted, TQIP lacks the granularity to distinguish these individuals from those admitted to a nonmedical facility such as a jail. Thus, we considered all individuals with this disposition of other facility to have been not admitted. Those who died in the ED were excluded from the final analysis of hospital admission.

### Covariates

TQIP contained demographic, clinical, and hospital data. Demographic variables included in this study were sex, age, race, ethnicity, and insurance status. Race and ethnicity were characterized using separate race and ethnicity variables as Hispanic, non-Hispanic Black, non-Hispanic White, and other (ie, American Indian, Asian, and Pacific Islander per the National Trauma Databank definitions). Race and ethnicity data in TQIP are based on self-report or report of a family member.^21^ Racial and ethnic disparities in admission have been widely documented elsewhere^22^; therefore, we controlled for race and ethnicity in the current study. Clinical comorbidities in TQIP include standard Elixhauser physical and behavioral health comorbidities.^23^ Physical health comorbidities in this study were heart disease, hypertension, chronic obstructive pulmonary disease, chronic kidney disease, diabetes, malignant neoplasms, and liver disease. Behavioral health comorbidities included schizophrenia, bipolar disorder, major depressive disorder, social anxiety disorder, posttraumatic stress disorder, and antisocial personality disorder.^21^ Injury characteristics included alcohol and drug positivity, trauma type (blunt, penetrating, or other), mechanism, intent (unintentional, self-inflicted, assault, or other), body region, Injury Severity Score (ISS), and Glasgow Coma Scale (GCS) score. Injury body region was identified using *International Statistical Classification of Diseases and Related Health Problems, Tenth Revision *diagnosis codes in accordance with the Injury Mortality Diagnosis Matrix.^24^ ISS and GCS scores were grouped into clinically meaningful categories (ISS: minor injury, 1-8; moderate, 9-15; and severe, ≥16; GCS score: severe head injury, 3-8; moderate, 9-12; and minor, 13-15).^25,26^ Trauma center designation was determined using ACS verification level, and state designation was used when verification was unavailable. When hospital-level data were known for some encounters and missing for others in the same hospital, missing hospital variable data were imputed under the assumption that hospital-level data would be the same for all encounters at a given hospital. Missing patient-level data were not imputed.

### Statistical Analysis

Data were analyzed from December 2021 to November 2022. ED discharge disposition was compared between PEH and housed patients using χ^2^ tests. We then fitted hierarchical multivariable logistic regression models with hospital-level random intercepts to assess the association between being unhoused and odds of admission. These models controlled for age, race, insurance, trauma type, GCS score, intent, and ISS. All tests were 2-sided with α < .05 indicating statistical significance. Analyses were performed using Stata MP statistical software version 17.0 (StataCorp).

Two separate subgroup analyses were performed to reduce the effect of confounding. The first was an analysis of PEH propensity score–matched to all housed patients.^27^ Details of the matching process are described in the eMethods in Supplement 1.^28^ PEH and housed patients were matched on sex, age, insurance, injury type, body region, and physical and/or behavioral health comorbidity. Hospital identity was used as an exact matching criterion to account for hospital-level differences in admission practices. Postmatch characteristics were compared using standardized differences and showed nearly complete matching of PEH with better balanced distributions of demographic and clinical (eTable 1 in Supplement 1) as well as injury characteristics (eTable 2 in Supplement 1). Stratified analysis compared differences in admission rates among matched pairs across ISS with McNemar tests. Hierarchical logistic regression clustering by both matched pairs and hospitals evaluated the association between homelessness and odds of admission.

The second subgroup analysis evaluated differences between PEH and low-income housed patients to account for socioeconomic status as a potential confounder. Demographic and injury characteristics were compared with χ^2^ tests. Unadjusted admission rates were compared between groups. Hierarchical multivariable logistic regression with hospital-level random effects was used to assess associations between homelessness and admission. These models controlled for age, race, trauma type, GCS score, intent, and ISS. Subgroup analyses were hypothesis generating; results were not adjusted for multiple testing.

## Results

There were 1 738 992 patient encounters who presented to 790 hospitals (mean [SD] age, 53.6 [21.2] years; 712 120 [41.0%] female; 97 910 [5.9%] Hispanic, 227 638 [13.7%] non-Hispanic Black, and 1 157 950 [69.6%] non-Hispanic White). Of these, 12 266 (0.7%) were PEH. Compared with housed patients, PEH were younger (mean [SD] age, 45.2 [13.6] years vs 53.7 [21.3] years), were more often male (10 343 patients [84.3%] vs 1 016 310 patients [58.9%]), non-Hispanic Black (2595 patients [21.2%] vs 225 043 patients [13.0%]), and insured by Medicaid (5918 patients [48.3%] vs 237 692 patients [13.8%]) (Table 1). PEH also exhibited higher incidence of substance use disorder (5480 patients [44.7%] vs 180 922 patients [10.5%]) and behavioral comorbidity (2884 patients [23.5%] vs 191 425 patients [11.1%]) than housed patients.

**Table 1.** Demographic and Clinical Characteristics of Injured PEH and All Housed Patients

Characteristics	Patients, No. (%)		*P* value^a^
	PEH (n = 12 266 [0.7%])	Housed (n = 1 726 726 [99.3%])	
Sex			
Male	10 343 (84.3)	1 016 310 (58.9)	<.001
Female	1921 (15.7)	710 199 (41.1)	
Missing	2 (0.02)	217 (0.01)	NA
Age, y			
18-35	3467 (28.3)	429 756 (25.9)	<.001
36-50	3825 (31.2)	273 859 (15.9)	
51-64	4164 (33.9)	324 669 (18.8)	
≥65	764 (6.2)	575 864 (33.3)	
Missing	46 (0.4)	122 578 (7.1)	NA
Race and ethnicity			
Hispanic	1254 (10.2)	96 656 (5.6)	<.001
Non-Hispanic Black	2595 (21.2)	225 043 (13.0)	
Non-Hispanic White	6191 (50.5)	1 151 759 (66.7)	
Other^b^	1903 (15.5)	178 793 (10.4)	
Missing	323 (2.6)	74 475 (4.3)	NA
Insurance			
Private	1434 (11.7)	567 248 (32.8)	<.001
Uninsured	2851 (23.2)	187 774 (10.9)	
Medicaid	5918 (48.3)	237 692 (13.8)	
Medicare	1240 (10.1)	600 599 (34.8)	
Other	766 (6.2)	99 219 (5.7)	
Missing	57 (0.5)	34 194 (2.0)	NA
Comorbidities			
Any physical comorbidity^c^	4138 (33.7)	912 635 (52.8)	<.001
Substance use disorder	5480 (44.7)	180 922 (10.5)	<.001
Any behavioral comorbidity^d^	2884 (23.5)	191 425 (11.1)	<.001
Trauma center			
Level I	7400 (60.3)	866 234 (50.2)	<.001
Level II	4245 (34.6)	628 464 (36.4)	
Nontrauma	574 (4.7)	217 529 (12.6)	
Missing	47 (0.4)	14 499 (0.8)	NA
Teaching status			
Community	4624 (37.7)	678 086 (39.3)	<.001
Nonteaching	1115 (9.1)	309 828 (17.9)	
University	6520 (53.2)	733 782 (42.5)	
Missing	7 (0.1)	5030 (0.3)	NA

Abbreviations: NA, not applicable; PEH, people experiencing homelessness.

^a^ *P* values were derived from χ^2^ tests of independence.

^b^ Refers to American Indian, Asian, and Pacific Islander.

^c^ Physical comorbidities include heart disease, hypertension, chronic obstructive pulmonary disease, chronic kidney disease, diabetes, malignant neoplasm, or liver disease.

^d^ Behavioral comorbidities include schizophrenia, bipolar disorder, major depressive disorder, social anxiety disorder, posttraumatic stress disorder, and antisocial personality disorder.

### Injury Characteristics of PEH

PEH more often tested positive for alcohol (3982 patients [32.5%] vs 224 891 patients [13.0%]) and drugs (4599 patients [37.5%] vs 212 582 patients [12.3%]) than housed patients, although they were also screened more often (Table 2). Compared with housed patients, PEH more often sustained penetrating (2236 patients [18.2%] vs 150 431 patients [8.7%]) and pedestrian-strike (1891 patients [15.4%] vs 55 533 patients [3.2%]) injuries. Assault accounted for 36.0% of injuries (4417 patients) among PEH and 9.6% of injuries (165 666 patients) among housed patients. Injuries to the head and neck were more common among PEH than housed patients (8041 patients [65.6%] vs 851 823 patients [49.3%]). PEH were also more likely than housed patients to present with ISS greater than or equal to 16 (2396 patients [19.5%] vs 279 129 patients [16.2%]) and severe traumatic brain injury (GCS score 3-8, 928 patients [7.6%] vs 84 675 patients [4.9%]). Compared with nontrauma centers, level I and II trauma centers significantly more often treated PEH with pedestrian-strike injury (1091 patients [15.7%] vs 729 patients [18.6%] vs 62 patients [11.5%]) and less often saw PEH injured from falls (1777 patients [25.6%] vs 988 patients [25.3%] vs 180 patients [33.3%]).

**Table 2.** Injury Characteristics of Injured PEH and All Housed Patients

Injury characteristics	Patients, No. (%)		*P* value^a^
	PEH (n = 12 266 [0.7%])	Housed (n = 1 726 726 [99.3%])	
Alcohol use			
Negative	5358 (43.7)	575 598 (33.3)	<.001
Positive	3982 (32.5)	224 891 (13.0)	
Not tested	2861 (23.3)	886 941 (51.4)	
Missing	65 (0.5)	39 296 (2.3)	NA
Drug use			
Negative	2292 (18.7)	288 281 (16.7)	<.001
Positive	4599 (37.5)	212 582 (12.3)	
Not tested	5201 (42.4)	1 163 982 (67.4)	
Missing	174 (1.4)	61 881 (3.6)	NA
Trauma type			
Blunt	9123 (74.4)	1 512 120 (87.6)	<.001
Penetrating	2236 (18.2)	150 431 (8.7)	
Other	607 (5.0)	43 750 (2.5)	
Missing	300 (1.2)	20 425 (1.2)	NA
Mechanism			
Fall	2952 (24.1)	831 653 (48.2)	<.001
Cut or stabbing	1554 (12.7)	73 040 (4.2)	
Firearm	632 (5.2)	70 639 (4.1)	
Struck by or against	2371 (19.3)	102 990 (6.0)	
Motor vehicle collision	974 (7.9)	416 578 (24.1)	
Other transport	364 (3.0)	51 207 (3.0)	
Pedestrian struck	1891 (15.4)	55 533 (3.2)	
Other	707 (5.8)	92 764 (5.4)	
Missing	821 (6.7)	32 422 (1.9)	NA
Intent			
Unintentional	7027 (57.3)	1 511 281 (87.5)	<.001
Self-inflicted	387 (3.2)	24 092 (1.4)	
Assault	4417 (36.0)	165 666 (9.6)	
Other	358 (2.9)	12 312 (0.7)	
Missing	77 (0.6)	13 375 (0.8)	NA
Injury body region^b^			
Head or neck	8041 (65.6)	851 823 (49.3)	<.001
Spine	1870 (15.2)	314 712 (18.2)	<.001
Torso	4798 (39.1)	598 908 (34.7)	<.001
Extremity	6674 (54.4)	1 042 600 (60.4)	<.001
Missing	94 (0.8)	13 133 (0.7)	NA
Injury Severity Score			
Mild injury: 1-8	6241 (50.9)	832 926 (48.2)	<.001
Moderate injury: 9-15	3604 (29.4)	610 060 (35.3)	
Severe injury: ≥16	2396 (19.5)	279 129 (16.2)	
Missing	25 (0.2)	4611 (0.3)	NA
Initial Glasgow Coma Scale score			
3-8	928 (7.6)	84 675 (4.9)	<.001
9-12	665 (5.4)	37 641 (2.2)	
13-15	10 256 (83.6)	1 522 071 (88.1)	
Missing	417 (3.4)	82 339 (4.8)	NA

Abbreviations: NA, not applicable; PEH, people experiencing homelessness.

^a^ *P* values were derived from χ^2^ tests of independence.

^b^ Patients may present with injury to more than 1 body region.

### ED Discharge Disposition and Hospital Admission

Unadjusted rates of discharge home were similar between PEH and housed patients (1185 patients [9.7%] vs 159 385 patients [9.2%]), although PEH were more often admitted to an observation unit (895 patients [7.3%] vs 63 978 patients [3.7%]) (Table 3). Rates of death in the ED were comparable between groups (57 patients [0.5%] vs 6485 patients [0.4%]). PEH and housed patients demonstrated similar unadjusted rates of admission (10 888 patients [89.2%] vs 1 554 353 patients [90.4%]). On multivariable analysis, PEH had an associated 33.1% increased odds of admission (aOR, 1.33; 95% CI, 1.24-1.43; *P* < .001) (Table 4) compared with housed patients when controlling for age, race, insurance, trauma type, GCS score, intent, and ISS.

**Table 3.** Unadjusted Emergency Department Discharge Disposition Rates of Injured PEH and All Housed Patients

Disposition	Patients, No. (%)^a^	
	PEH (n = 12 266 [0.7%])	Housed (n = 1 726 726 [99.3%])
Observation unit	895 (7.3)	63 978 (3.7)
Inpatient unit	9799 (79.9)	1 435 888 (83.2)
Transferred	194 (1.6)	54 487 (3.2)
Home	1185 (9.7)	159 385 (9.2)
Other facility^b^	136 (1.1)	6503 (0.4)
Deceased	57 (0.5)	6485 (0.4)

Abbreviation: PEH, People experiencing homelessness.

^a^ *P* < .001 for all comparisons (χ^2^ test of independence).

^b^ Other facility includes jail, institutional care, and mental health facilities.

**Table 4.** Multivariable Model for Hospital Admission in Injured People Experiencing Homelessness and All Housed Patients

Characteristics	OR (95% CI)	*P* value^a^
Housing status		
Housed	1 [Reference]	NA
People experiencing homelessness	1.33 (1.24-1.43)	<.001
Age, y		
18-35	1 [Reference]	NA
36-50	1.24 (1.22-1.26)	<.001
51-64	1.65 (1.62-1.68)	<.001
≥65	2.17 (2.11-2.22)	<.001
Race and ethnicity		
Hispanic	0.93 (0.91-0.96)	<.001
Non-Hispanic Black	0.93 (0.91-0.95)	<.001
Non-Hispanic White	1 [Reference]	NA
Other^b^	1.08 (1.05-1.10)	<.001
Insurance		
Private	1 [Reference]	NA
Uninsured	0.72 (0.70-0.73)	<.001
Medicaid	1.24 (1.22-1.27)	<.001
Medicare	1.38 (1.35-1.42)	<.001
Other	0.96 (0.94-0.99)	.01
Trauma type		
Blunt	1 [Reference]	NA
Penetrating	0.89 (0.87-0.91)	<.001
Other	1.17 (1.13-1.22)	<.001
Intent		
Unintentional	1 [Reference]	NA
Self-inflicted	1.42 (1.35-1.51)	<.001
Assault	0.79 (0.77-0.81)	<.001
Other or unspecified	0.98 (0.91-1.06)	.67
Initial Glasgow Coma Scale score		
3-8	1 [Reference]	NA
9-12	0.34 (0.31-0.37)	<.001
13-15	0.19 (0.18-0.21)	<.001
Injury Severity Score		
1-8	1 [Reference]	NA
9-15	9.64 (9.43-9.84)	<.001
≥16	38.0 (36.0-40.1)	<.001

Abbreviations: NA, not applicable; OR, odds ratio.

^a^ ORs and 95% CIs are estimated from a hierarchical logistic regression model allowing for clustering between hospitals.

^b^ Refers to American Indian, Asian, and Pacific Islander.

### Subgroup Analyses

#### PEH Compared With Propensity-Matched All Housed Patients

There were 12 148 propensity-matched pairs in 401 hospitals. Matched PEH demonstrated significantly higher rates of admission (10 791 patients [89.2%] vs 10 456 patients [86.5%]) compared with housed patients. Differences in admission rates were greatest for those with the least severe injuries (ISS 1-8, 2774 patients [78.8%] vs 2641 patients [75.0%]). Among the 3521 pairs with ISS 1 to 8, 545 (15.5%) demonstrated discordance in which the PEH was admitted while the matched housed counterpart was not. As ISS increased, rates of hospital admission increased, and the magnitude of difference between PEH and housed cohorts decreased (Figure). On hierarchical analysis, matched PEH had an associated 36.2% increased odds of admission (aOR, 1.36; 95% CI, 1.25-1.59; *P* < .001) compared with housed patients.

**Figure.**
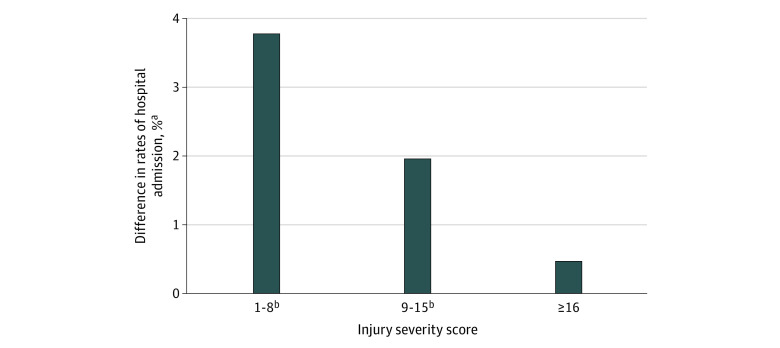
Differences in Rates of Admission Between Injured People Experiencing Homelessness (PEH) and Propensity-Matched All Housed Patients by Injury Severity Score ^a^Rates were calculated for matched pairs in which both the PEH and housed patients had an Injury Severity Score of the same category. ^b^Denotes groups for which differences in rates of admission were found to be statistically significant (*P* < .05) on McNemar tests.

#### PEH Compared With Low-Income Housed Patients

Subgroup analysis compared 12 266 PEH with 237 692 low-income housed patients. Compared with low-income housed patients, PEH were more often injured due to assault (4417 patients [36.0%] vs 58 716 patients [24.7%]) (eTable 3 in Supplement 1). Rates of penetrating injury were similar (2236 patients [18.2%] vs 43 898 patients [18.5%]), although PEH were more commonly injured by cuts or stabbings (1554 patients [12.7%] vs 20 304 patients [8.5%]), and low-income housed patients demonstrated higher rates of firearm injury (632 patients [5.2%] vs 22 137 patients [9.3%]). Unadjusted admission rates were slightly higher among PEH (10 888 patients [89.2%] vs 209 741 patients [88.5%]). On multivariable analysis, PEH demonstrated an associated 10.5% increased odds of admission (aOR, 1.10; 95% CI, 1.03-1.19; *P* < .001) (eTable 4 in Supplement 1) compared with low-income housed patients when controlling for age, race, trauma type, GCS score, intent, and ISS.

## Discussion

More than 500 000 people experience homelessness each night in the US.^1^ To our knowledge, this cohort study is the first nationwide study of traumatic injury among PEH. We found that PEH more frequently sustained pedestrian-strike injury and assault than housed patients. PEH were significantly more likely to be admitted to the hospital compared with both all housed patients and low-income housed patients. Differences in admission rates were greatest in cases of minor injury, potentially when disposition is influenced by clinical discretion.

Our findings of injury characteristics among PEH build on other single-center studies.^29^ Structural variables, such as lack of affordable housing, are important factors associated with homelessness, and vulnerabilities such as mental illness and substance use disorder are well documented individual-level risk factors for both homelessness and traumatic injury.^30,31,32,33^ Prior studies have demonstrated that burns and exposure-related injury were common among PEH.^34,35,36^ However, these studies were single center or city focused. Exposure to different mechanisms of injury is heavily influenced by local factors, such as weather, traffic, recreational activities, and local laws. Thus, results from a single center or city cannot be generalizable to other areas.

Our study is the first to elucidate national injury patterns among PEH. Similar to the high rates of assault in our study, Kushel et al^37^ found that up to 30% of PEH in San Francisco experience intentional physical assault. Factors that increase a PEH’s risk of assault include lack of protective shelter, proximity to high-crime areas, and substance use.^38,39,40^ Being a target of violence has been associated with increased ED use and poor health.^14,41^ In addition, homicides are a common cause of death among unhoused young adults.^9,13,42^ Our findings can be used to inform injury prevention initiatives for PEH.

Although the association between homelessness and hospitalization has been shown for other diagnoses, it has not been demonstrated in trauma patients.^4,29^ Reasons for increased adjusted odds of admission of injured PEH were likely multifactorial. A major factor may be clinicians’ perceived risks of discharging an unhoused person back to the street. Qualitative studies have shown that ED clinicians consider safety concerns when deciding to admit PEH with low medical acuity.^43^ The persistence of increased odds of admission among PEH compared with low-income housed patients suggests that the observed associations are due to unstable housing independently of other health-related social needs. For many PEH, discharge home would mean returning to the place where they were injured, potentially putting them at risk for reinjury. Furthermore, wound care and follow-up can be challenging in unstable housing conditions.^6,44^

### Implications

Our findings have several implications. Injury prevention efforts among PEH must be tailored to the unique injury patterns demonstrated, particularly the striking vulnerability to assault. High rates of admission suggest that hospitals are acting as social safety nets for injured PEH. Hospitals disproportionately admit PEH, likely recognizing the challenges of recovering and preventing reinjury in unstable housing. Admitted PEH may receive needed health and social service resource referrals.^45^ Our results underscore hospitals’ current role in providing wraparound social care services. This has important implications given the rising cost of health care as well as the inability of most acute care hospitals to provide such services to patients once they return to the community.

### Limitations

This study has several limitations. First, TQIP contains records from mostly level I and II trauma centers, and results may not be generalizable to PEH presenting to nonspecialized centers. However, the inclusion of nontrauma centers improves the generalizability of our findings. Similarly, records in TQIP demonstrate high rates of admission, and results may not be generalizable to hospitals that see more minor injuries and admit a lower proportion of injured patients. However, it is likely that differences between PEH and housed patients would be more pronounced in hospitals with greater variation in admission practices. Selection bias may have been introduced in the severity of injury captured, as well as clinician bias to admit patients. Nonetheless, we conducted multivariable regression and propensity score matching to control for variables that could affect bias in admission. Second, hospital and state policy may affect whether discharge home from the ED is permissible for PEH. However, we accounted for this with the use of hospital ID as an exact matching criterion in the propensity-matched analysis. Third, the alternate home residence variable used to identify PEH was completed when patients’ ZIP code of primary residence was unknown. This variable would not capture patients who were temporarily unhoused or those whose documents listed a shelter, former residence, or home of a family member. This could have led to misclassification bias and likely underestimated the prevalence of PEH. However, demographic characteristics of patients identified as PEH were similar to those described in other studies, suggesting specificity of our PEH cohort.^33,36^ The alternate home residence variable likely identifies those who chronically experience homelessness. Fourth, TQIP does not contain socioeconomic status or income data, which are known factors associated with injury mechanism and outcomes.^46,47^ We addressed this by conducting a subanalysis of a low-income cohort as defined by Medicaid insurance, because eligibility is largely determined according to an individual’s Modified Adjusted Gross Income.^19,20^ Fifth, TQIP’s ED discharge disposition category of other facility included a wide range of facilities, such as jail and mental health facilities. We considered individuals with this disposition to not have been admitted. This disposition was more common among PEH, likely because a larger proportion of PEH were admitted to psychiatric care.^48^ Thus, we may be underestimating the percentage of PEH who received medical care, including psychiatric care, after injury.

## Conclusions

This national cohort study demonstrated that PEH are more likely than housed individuals to experience assault and pedestrian-strike injuries. Injury prevention efforts among PEH must be tailored to these unique injury patterns. PEH had increased adjusted odds of hospital admission after injury. These findings underscore potential opportunities for policy and social programming initiatives to improve the care and hospital use of injured PEH.

## Notes

Supplement 1. **eFigure.** Patient Selection Schema **eMethods.** Propensity Score Matching Methods **eTable 1.** Demographic and Clinical Characteristics of the Matched Cohort of People Experiencing Homelessness and All Housed Patients **eTable 2.** Injury Characteristics of the Matched Cohort of People Experiencing Homelessness and All Housed Patients **eTable 3.** Injury Characteristics of Injured People Experiencing Homelessness and Low-Income Housed Patients **eTable 4.** Multivariable Models for Hospital Admission in Injured People Experiencing Homelessness and Low-Income Housed Patients

Supplement 2.
Data Sharing Statement
