# Supplementary material for: Injury Patterns and Hospital Admission After Trauma Among People Experiencing Homelessness
Source: JAMA Netw Open. 2023 Jun 29;6(6):e2320862. doi: 10.1001/jamanetworkopen.2023.20862 (PMC10311388; doi:10.1001/jamanetworkopen.2023.20862)
Supplement: Supplement 1. — eFigure. Patient Selection Schema eMethods. Propensity Score Matching Methods eTable 1. Demographic and Clinical Characteristics of the Matched Cohort of People Experiencing Homelessness and All Housed Patients eTable 2. Injury Characteristics of the Matched Cohort of People Experiencing Homelessness and All Housed Patients eTable 3. Injury Characteristics of Injured People Experiencing Homelessness and Low-Income Housed Patients eTable 4. Multivariable Models for Hospital Admission in Injured People Experiencing Homelessness and Low-Income Housed Patients [file jamanetwopen-e2320862-s001.pdf]

Supplemental Online Content

Silver CM, Thomas AC, Reddy S, et al. Injury patterns and hospital admission after trauma among people experiencing homelessness. *JAMA Netw Open*. 2023;6(6):e2320862. doi:10.1001/jamanetworkopen.2023.20862

eFigure. Patient Selection Schema

eMethods. Propensity Score Matching Methods

eTable 1. Demographic and Clinical Characteristics of the Matched Cohort of People Experiencing Homelessness and All Housed Patients

eTable 2. Injury Characteristics of the Matched Cohort of People Experiencing Homelessness and All Housed Patients

eTable 3. Injury Characteristics of Injured People Experiencing Homelessness and Low-Income Housed Patients

eTable 4. Multivariable Models for Hospital Admission in Injured People Experiencing Homelessness and Low-Income Housed Patients

This supplemental material has been provided by the authors to give readers additional information about their work.

© 2023 Silver CM et al. *JAMA Network Open.*

eFigure. Patient Selection Schema

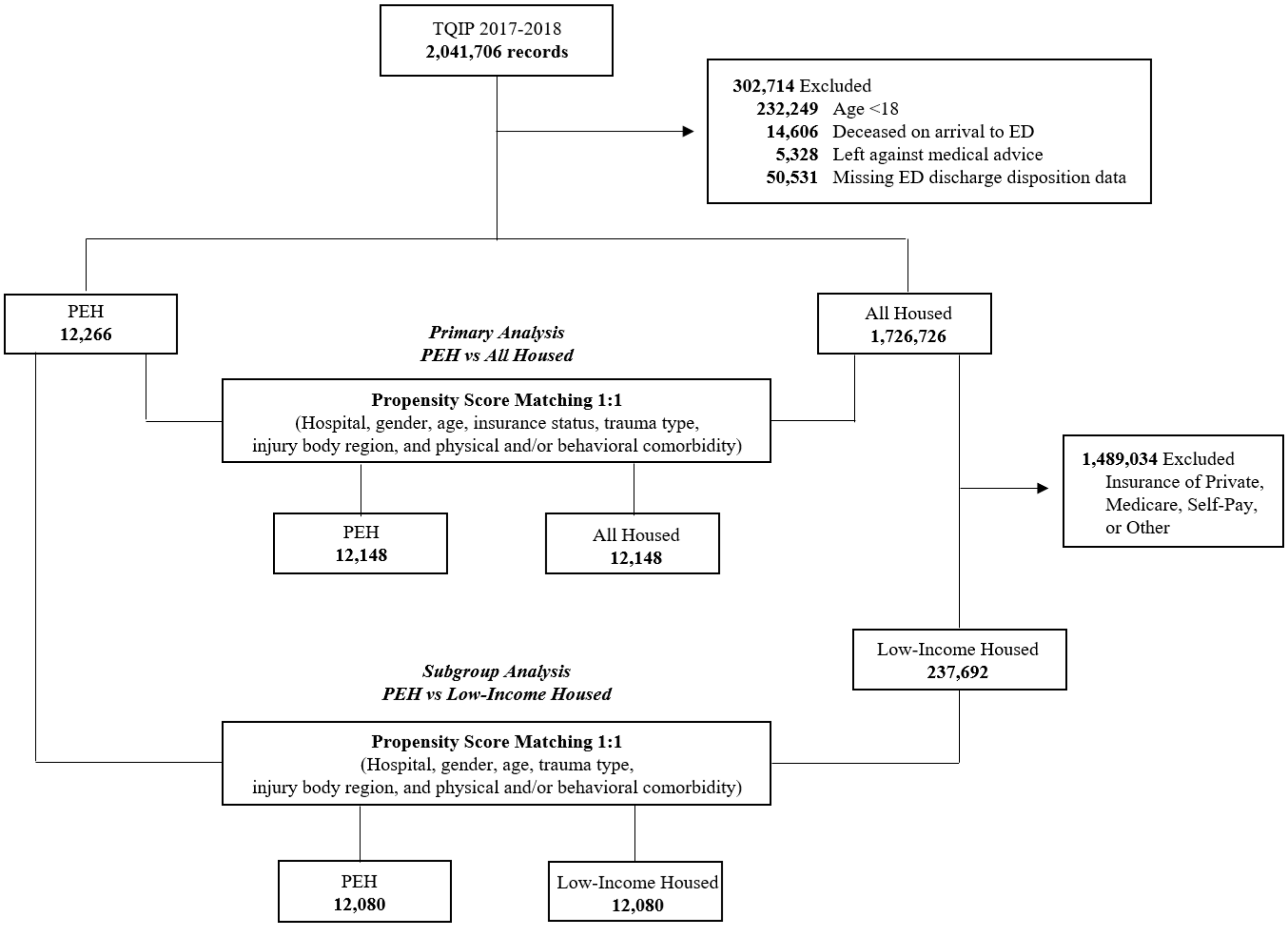

Abbreviations: PEH people experiencing homelessness

© 2023 Silver CM et al. *JAMA Network Open.*

eMethods. Propensity Score Matching Methods

A propensity score model predicting the probability of being a PEH was created using the Stata package psmatch2. This model adjusted for gender, age, insurance, injury type, body region, and the presence of any physical and/or behavioral health comorbidity. Hospital identity was used as an additional exact-matching criterion to account for differences in admission practices among hospitals. Propensity score matching was performed using the 1:1 greedy nearest neighbor algorithm with a maximum specified caliper width of 0.5.

© 2023 Silver CM et al. *JAMA Network Open.*

eTable 1. Demographic and Clinical Characteristics of the Matched Cohort of People Experiencing Homelessness and All Housed Patients

Characteristics	Housing Status, No. (%)		*Standardized Difference*[a]
	**PEH** N=12,148 (50.0%)	**Housed** N=12,148 (50.0%)	
Gender			
Male	10,253 (84.4)	10,332 (85.1)	<0.02[d]
Female	1,895 (15.6)	1,816 (14.9)	
Age			
18-35	3,434 (28.3)	3,680 (30.3)	<0.07[d]
36-50	3,804 (31.3)	3,647 (30.0)	
51-64	4,150 (34.2)	3,904 (32.1)	
≥ 65	760 (6.3)	917 (7.5)	
Race/Ethnicity			
Non-Hispanic White	6,141 (51.9)	5,722 (48.2)	<0.14
Non-Hispanic Black	2,567 (21.7)	2,275 (19.2)	
Hispanic	1,246 (10.5)	1,493 (12.6)	
Other	1,884 (15.9)	2,374 (20.0)	
Insurance			
Private	1,430 (11.8)	1,684 (13.9)	<0.10[d]
Uninsured	2,836 (23.4)	2,685 (22.1)	
Medicaid	5,895 (48.5)	5,526 (45.5)	
Medicare	1,230 (10.1)	1,345 (11.1)	
Other	757 (6.2)	908 (7.5)	
Comorbidities			
Any physical comorbidity[b]	4,105 (33.8)	4,036 (33.2)	0.01[d]
Substance use disorder	5,441 (44.8)	2,525 (20.8)	0.53
Behavioral comorbidity	2,864 (23.6)	2,721 (22.4)	0.03[d]
Trauma Center			
Level I	7,355 (60.7)	7,346 (60.7)	0.002
Level II	4,198 (34.7)	4,210 (34.8)	
Non-Trauma	557 (4.6)	554 (4.6)	
Teaching Status			
Community	4,572 (37.7)	4,580 (37.7)	0.001
Non-Teaching	1,101 (9.1)	1,101 (9.1)	
University	6,468 (53.3)	6,460 (53.2)	

Abbreviations: PEH People Experiencing Homelessness

[a] Standardized differences are equal to the absolute value of the difference in proportions divided by the standard error. It is considered an indicator of effect size: >0.20 indicates small, >0.50 medium, and ≥0.80 large effect size

[b] Medical comorbidities include heart disease, hypertension, chronic obstructive pulmonary disease, chronic kidney disease, diabetes, malignancy, or liver disease

[c] Behavioral comorbidities include schizophrenia, bipolar disorder, major depressive disorder, social anxiety disorder, posttraumatic stress disorder, and antisocial personality disorder

[d] Characteristics on which patients were propensity matched

© 2023 Silver CM et al. *JAMA Network Open.*

eTable 2. Injury Characteristics of the Matched Cohort of People Experiencing Homelessness and All Housed Patients

Injury Characteristics	Housing Status, No. (%)		Standardized Difference[a]
	**PEH** N=12,148 (50.0%)	**Housed** N=12,148 (50.0%)	
Alcohol Use			
Negative	5,318 (44.0)	5,198 (43.0)	
Positive	3,956 (32.7)	3,196 (26.5)	0.18
Not Tested	2,815 (23.3)	3,686 (30.5)	
Drug Use			
Negative	2,266 (18.9)	2,570 (21.5)	
Positive	4,566 (38.1)	2,940 (24.6)	0.30
Not Tested	5,146 (43.0)	6,435 (53.9)	
Trauma Type			
Blunt	9,032 (74.4)	9,095 (74.9)	
Penetrating	2,218 (18.3)	2,232 (18.4)	0.02[b]
Other	898 (7.4)	821 (6.8)	
Mechanism			
Fall	3,353 (28.9)	2,915 (25.7)	
Cut/Stabbing	1,271 (10.9)	1,541 (13.6)	
Firearm	916 (7.9)	628 (5.5)	
Struck by/against	1,447 (12.5)	2,351 (20.8)	0.56
MVC	2,686 (23.1)	962 (8.5)	
Other Transport	426 (3.7)	363 (3.2)	
Pedestrian Struck	741 (6.4)	1,880 (16.6)	
Other	767 (6.6)	691 (6.1)	
Intent			
Unintentional	6,957 (57.6)	8,344 (69.4)	
Self-Inflicted	381 (3.2)	445 (3.7)	0.27
Assault	4,376 (36.2)	2,920 (24.3)	
Other/unspecified	358 (3.0)	305 (2.5)	
Injury Body Region[b]			
Head or Neck	4,809 (39.6)	4,838 (39.8)	
Spine	687 (5.7)	663 (5.5)	0.03[b]
Torso	4,767 (39.2)	4,782 (39.4)	
Extremity	1,788 (14.7)	1,791 (14.7)	
System-Wide	97 (0.8)	74 (0.6)	
ISS			
Mild injury: 1-8	6,175 (50.9)	6,255 (51.6)	
Moderate injury: 9-12	3,575 (29.5)	3,586 (29.6)	0.02
Severe injury: ≥16	2,374 (19.6)	2,288 (18.9)	
Initial GCS			
3-8	911 (7.8)	895 (7.6)	
9-12	659 (5.6)	438 (3.7)	0.09
13-15	10,168 (86.6)	10,462 (88.7)	

Abbreviations: PEH people experiencing homelessness, MVC motor vehicle collision, ISS injury severity score, GCS Glasgow coma scale

[a] Standardized differences are equal to the absolute value of the difference in proportions divided by the standard error. It is considered an indicator of effect size: >0.20 indicates small, >0.50 medium, and ≥0.80 large effect size

[b] Characteristics on which patients were propensity matched

© 2023 Silver CM et al. *JAMA Network Open.*

eTable 3. Injury Characteristics of Injured People Experiencing Homelessness and Low-Income Housed Patients

Injury Characteristics	Housing Status, No. (%)		p-value[a]
	PEH N=12,266 (4.9%)	Low-Income Housed N=237,692 (95.1%)	
Alcohol Use			
Negative	5,358 (43.7)	94,294 (39.7)	<0.001
Positive	3,982 (32.5)	52,437 (22.1)	
Not Tested	2,861 (23.3)	87,213 (36.7)	
Missing	65 (0.5)	3,748 (1.6)	
Drug Use			
Negative	2,292 (18.7)	40,415 (17.0)	<0.001
Positive	4,599 (37.5)	60,170 (25.3)	
Not Tested	5,201 (42.4)	128,801 (54.2)	
Missing	174 (1.4)	8,306 (3.5)	
Trauma Type			
Blunt	9,123 (74.4)	178,630 (75.2)	<0.001
Penetrating	2,236 (18.2)	43,898 (18.5)	
Other	607 (5.0)	10,520 (4.4)	
Missing	300 (1.2)	4,644 (1.9)	
Mechanism			
Fall	2,952 (24.1)	70,194 (29.5)	<0.001
Cut/Stabbing	1,554 (12.7)	20,304 (8.5)	
Firearm	632 (5.2)	22,137 (9.3)	
Struck by/against	2,371 (19.3)	26,463 (11.1)	
MVC	974 (7.9)	54,666 (23.0)	
Other Transport	364 (3.0)	6,888 (2.9)	
Pedestrian Struck	1,891 (15.4)	11,796 (5.0)	
Other	707 (5.8)	18,417 (7.8)	
Missing	821 (6.7)	6,827 (2.9)	
Intent			
Unintentional	7,027 (57.3)	167,380 (70.4)	<0.001
Self-Inflicted	387 (3.2)	6,100 (2.6)	
Assault	4,417 (36.0)	58,716 (24.7)	
Other	358 (2.9)	3,413 (1.4)	
Missing	77 (0.6)	2,083 (0.9)	
Injury Body Region[b]			
Head or Neck	8,041 (65.6)	127,691 (53.7)	<0.001
Spine	1,870 (15.2)	38,425 (16.2)	0.01
Torso	4,798 (39.1)	89,552 (37.7)	0.001
Extremity	6,674 (54.4)	136,062 (57.2)	<0.001
Missing	94 (0.8)	2,313 (1.0)	
ISS			
Mild injury: 1-8	6,241 (50.9)	125,108 (52.8)	<0.001
Moderate injury: 9-12	3,604 (29.4)	69,430 (29.3)	
Severe injury: ≥16	2,396 (19.5)	42,367 (17.9)	
Missing	25 (0.2)	787 (0.3)	
Initial GCS			
3-8	928 (7.6)	16,415 (7.2)	<0.001
9-12	665 (5.4)	6,957 (3.1)	
13-15	10,256 (83.6)	203,237 (89.7)	
Missing	417 (3.4)	11,083 (4.7)	

Abbreviations: MVC motor vehicle collision, ISS injury severity score, GCS Glasgow coma scale

[a] P-values derived from chi-square tests of independence

[b] Patients may present with injury to more than one body region

© 2023 Silver CM et al. *JAMA Network Open.*

eTable 4. Multivariable Models for Hospital Admission in Injured People Experiencing Homelessness and Low-Income Housed Patients

Characteristics	OR (95% CI)	*p-value*[a]
Housing Status		
Housed	Reference	
PEH	1.10 (1.03-1.19)	0.01
Age		
18-35	Reference	
36-50	1.26 (1.22-1.31)	<0.001
51-64	1,71 (1.64-1.79)	<0.001
>= 65	2.01 (1.83-2.22)	<0.001
Race/Ethnicity		
Non-Hispanic White	Reference	
Non-Hispanic Black	0.95 (0.91-1.00)	0.04
Hispanic	0.89 (0.83-0.95)	<0.001
Other	1.21 (1.15-1.28)	<0.001
Trauma Type		
Blunt	Reference	
Penetrating	0.92 (0.88-0.96)	<0.001
Other	1.33 (1.23-1.45)	<0.001
Intent		
Unintentional	Reference	
Self-Inflicted	1.37 (1.23-1.53)	<0.001
Assault	0.82 (0.79-0.86)	<0.001
Other/unspecified	0.99 (0.86-1.14)	0.91
Initial GCS		
3-8	Reference	
9-12	0.33 (0.27-0.41)	<0.001
13-15	0.18 (0.15-0.22)	<0.001
ISS		
1-8	Reference	
9-15	10.22 (9.68-10.78)	<0.001
>=16	47.3 (41.0-54.6)	<0.001

Abbreviations: OR odds ratio, CI confidence interval, PEH people experiencing homelessness, GCS Glasgow coma score, ISS injury severity score

[a] ORs and CIs are estimated from a hierarchical logistic regression model allowing for clustering between hospitals

© 2023 Silver CM et al. *JAMA Network Open.*
