# Supplementary material for: Injury Patterns and Hospital Admission After Trauma Among People Experiencing Homelessness
Source: JAMA Netw Open. 2023 Jun 29;6(6):e2320862. doi: 10.1001/jamanetworkopen.2023.20862 (PMC10311388; doi:10.1001/jamanetworkopen.2023.20862)
Supplement: Supplement 2. — Data Sharing Statement [file jamanetwopen-e2320862-s002.pdf]

Data Sharing Statement

Silver. Injury Patterns and Hospital Admission After Trauma Among People Experiencing Homelessness. *JAMA Netw Open*. Published June 29, 2023.
doi:10.1001/jamanetworkopen.2023.20862

Data
Data available: No

Additional Information: Data reported in this study may be requested from the American College of Surgeons Trauma Quality Improvement Program through a data use agreement.
